# Accuracy of cross-sectional imaging in predicting tumor viability using the LI-RADS treatment response algorithm after image-guided percutaneous ablation with radiologic-pathologic explant correlation

**DOI:** 10.1186/s40644-025-00884-y

**Published:** 2025-05-24

**Authors:** Anuradha S. Shenoy-Bhangle, M. Saad Malik, Aamir Ali, Nan Nancy Jiang, Syed Yasir Andrabi, Amit Singal, Michael P. Curry, Maria-Andreea Catana, Devin E. Eckhoff, Salomao Faintuch, Muneeb Ahmed, Imad Ahmad Nasser, Ammar Sarwar

**Affiliations:** 1 Division of Abdominal Imaging, Massachusetts General Hospital, 55 Fruit Street, Boston, MA 02114 USA; 2 Department of Radiology, Beth Israel Deaconess Medical Center, 330 Brookline Avenue, Boston, MA 02215 USA; 3 Department of Internal Medicine, University of Texas Southwestern, 6202 Harry Hines Blvd, Dallas, TX 75235 USA; 4 Division of Gastroenterology/Liver Center, Beth Israel Deaconess Medical Center, 110 Francis Street, Boston, MA 02215 USA; 5 Department of Surgery, Beth Israel Deaconess Medical Center, 110 Francis Street, Boston, MA 02215 USA; 6 Department of Pathology, Beth Israel Deaconess Medical Center, 330 Brookline Avenue, Boston, MA 02215 USA

**Keywords:** HCC, Percutaneous ablation, LI-RADS treatment response, Explant, Pathology

## Abstract

**Background:**

Accurate assessment of viable HCC on pre-transplant cross sectional imaging is important for correct organ allocation and overall patient outcome following liver transplantation.

**Purpose:**

Determine accuracy of LI-RADS TRA compared to explant pathology in patients treated with thermal ablation, using contrast enhanced multiphase CT and MRI.

**Materials and methods:**

Imaging studies for 119 consecutive adult HCC patients treated with thermal ablation and liver transplantation from March 2001 to September 2019 at a single tertiary care hospital were retrospectively studied by three Board-certified radiologists. LI-RADS TRA categories for each tumor were compared with explant pathology. Cohens Kappa test and inter-reader agreement by Fleiss κ test, with 95% confidence intervals obtained with bootstrap technique were used.

**Results:**

Of the 119 patients (median age 59 years, 95 [80%] male) with 165 HCCs treated with percutaneous thermal ablation, 68% were completely necrotic and 32% were viable on pathologic analysis. Tumors viable on explant were larger on pre-treatment imaging (median 2.4 vs. 2.1 cm; *p* = 0.02) with no difference in pre-transplant ablation cavity sizes between groups (4.0 vs. 3.9 cm, respectively; *p* = 0.58). NPV of LI-RADS TRA for viable tumor was 71% (68–74); PPV of 62.5% (39–81) (*p* = 0.01) with a sensitivity of 19% (9.4–32), specificity of 95% (89–98), and accuracy of 70% (63–77). On explant, 55 incidental treatment naïve viable tumors, not visible on pre-transplant imaging, were found in 33 patients.

**Conclusion:**

The “non-viable” category of LI-RADS TRA even when applied to a relatively uniform percutaneous ablation cohort, demonstrated low sensitivity in predicting absence of viable tumor. MRI had more accuracy than CT in predicting tumor viability when compared to explant pathology.

**Supplementary Information:**

The online version contains supplementary material available at 10.1186/s40644-025-00884-y.

## Introduction

Liver transplantation (LT) for patients with early-stage hepatocellular carcinoma (HCC), as defined by the Milan criteria has consistently led to 5-year survival rates exceeding 70% [1]. Several single center studies demonstrated that achieving complete pathologic necrosis (CPN) with locoregional therapy (LRT) prior to LT is associated with improved outcomes after LT [2, 3]. More recently, a large US multicenter study consisting of 3,439 patients demonstrated that achieving complete pathologic necrosis was associated with lower post-transplant tumor recurrence and better 5-year survival [4]. Thus, accurate assessment of achieving CPN prior to LT has implications on outcomes after LT.

Pre-transplant imaging assessment for HCC is performed using the Liver Imaging Reporting and Data System (LI-RADS) algorithm which emphasizes specificity over sensitivity [5, 6]. Assessment of treatment response and CPN after locoregional therapy is performed using the LI-RADS treatment response algorithm (TRA/LR-TR categories) [7], which similarly has demonstrated high specificity ranging from 81 to 97% and an accuracy of 67–71% with pathologic correlation [8, 9]. However, a study by Hassan et al. [10] demonstrated a specificity of 92% (reader 1) and 69% (reader 2) of the LIRADS and TRA on a per-patient basis for detecting viable tumors on explant, suggesting some inter-reader variability. Another limitation of the LI-RADS TRA is that systematic reviews have found fewer than 5 high quality studies comparing it to pathology, most studies have a majority of patients treated using chemoembolization, pre-transplant imaging is mostly performed with magnetic resonance imaging (MRI) using hepatobiliary phase imaging, and interpretation is performed by experienced readers, generally with more than 9 years of experience [11- 13].

Moreover, recent studies in the UNOS database have shown that the median CPN rate on explant at transplant centers in the United States is 25%, due to under-use of curative therapies such as ablation but this may also be related to under-diagnosis (misdiagnosis of patients with viable tumor as having no viable tumor on imaging) of HCC in the transplant wait-list setting [14]. Therefore, the goal of our study was to determine accuracy of LI-RADS TRA after percutaneous ablation therapy correlated with explant pathology.

## Methods

This is a retrospective study performed after obtaining the hospital Institutional Review Board approval which waived the requirement for written informed consent. The study procedures conformed to all the requirements of the Health Insurance Portability and Accountability Act.

### Demographic data

The electronic patient database at a single tertiary care academic medical and transplant center was queried to identify consecutive adult patients with biopsy proven or imaging diagnosis of HCC, who underwent liver-directed therapy followed by LT between March 2001 and September 2019 (*n* = 187). Inclusion criteria included: (a) those that underwent image-guided percutaneous thermal ablation (either microwave or radiofrequency ablation); (b) those with pre and post treatment multiphase CT/MRI scans conforming to the technical requirements of LI-RADS (v2018). Of the patients undergoing MRI (*n* = 78), only those that received an extracellular contrast agent were included in the study as our center does not routinely use a hepatobiliary agent for routine post-treatment liver imaging. Exclusion criteria included those (a) patients without a multiphase cross-sectional exam, either before or after treatment or within 3 months of LT (*n* = 20), (b) imaging performed with a hepatobiliary MRI contrast agent (*n* = 2), or (c) patients treated with intra-arterial therapy (*n* = 46). (Figure S1).

Patient details collected for the study included age, gender, etiology of chronic liver disease, Eastern cooperative oncology group (ECOG) performance status, baseline comorbidities including presence of ascites and encephalopathy, baseline laboratory values, and Child-Pugh (CP) scores.

### Treatment method

There were 119 patients who underwent a total of 142 percutaneous image-guided thermal ablations prior to LT. All LRT procedures were performed by fellowship-trained interventional radiology faculty members. Radiofrequency ablation (RFA) was performed using the Cool Tip™ RF ablation system (Medtronic, Dublin, Ireland) with ablation parameters selected based on tumor size and manufacturer recommendations. Microwave ablation (MWA) was adopted at this center in 2017. MWA was performed using an AMICA system (HS Hospital Service, Roma, Italy) with ablation parameters selected based on tumor size and manufacturer recommendations. For both RFA and MWA, tumor localization and probe placement were accomplished using a combination of non-contrast CT and ultrasound (US) guidance. Decisions regarding the use of overlapping ablations were made intra-procedurally by the attending interventional radiologist, and the number of overlapping ablations were recorded. Intra-ablation fluoroscopy and immediate post procedure multiphasic CT was performed using iodinated contrast (Omnipaque 350, GE Healthcare) in all patients to evaluate the adequacy of the ablation zone.

### Image acquisition

All cross-sectional imaging studies, including multiphase CT or multiphase contrast enhanced MRI scans, were confirmed to have the necessary images as described in the standard liver imaging protocol which follows the LI-RADS technical requirement recommendation for diagnosis of HCC [15]. Multiphase CT was performed on a 64–detector row unit (Discovery CT750 HD; GE Medical Systems, Milwaukee, Wis) using iodinated intravenous contrast (Omnipaque 350, GE Healthcare) in late arterial, portal venous, and a 3-minute delayed phase to include the entire liver. MRI examinations were performed using a torso phased array coil on either a 1.5T (Magnetom Avanto or Aera, Siemens Healthcare) or 3T (Magnetom Trio or Skyra, Siemens Healthcare) MRI system. Scans were performed without and with the use of intravenous Gadolinium (Gadobuterol, Bayer Healthcare Pharmaceuticals, United States), an extracellular contrast agent (0.05 mmol/kg) using a power injection system (Spectris Solaris EP, Medrad, Pittsburgh, PA) at a rate of 2 mL/s followed by a 20 mL saline bolus flush. MRI image acquisition technique and parameters are further explained in Supplemental methods.

### Timing of scans

Imaging studies obtained at three time-points were analyzed. These included studies (both multiphase CT and MRI scans) obtained before the first treatment; studies obtained 1 month after treatment, and pre-transplant studies obtained within 3 months prior to LT.

### Image analysis

Three Board-certified radiologists with fellowship training in abdominal radiology with an experience of 9 years (A.S.S.B), and 1 year after fellowship (N.N.J and S.Y.A), reviewed the pre- and post-treatment studies independently. Any discrepancies were resolved by joint consensus read. Scans were evaluated for the date they were performed, scan quality, number, and tumor location of each observation using Couinaud’s segmental distribution [16]. If there were more than one tumors, they were recorded in descending order of Couinaud’s nomenclature, for example, a tumor in the Caudate lobe was labelled as tumor 1, followed by a tumor in segment II, III, IVA and so on. This technique ensured uniformity and accuracy of tumor identification among the three readers.

Per-lesion assessment of size was measured in two perpendicular dimensions relative to the maximum axial dimension, LI-RADS category was assigned for each lesion on the pre-treatment study including major features such as arterial phase hyperenhancement (APHE), washout, and capsule on delayed-phase imaging. LR-1 and LR-2 observations were not recorded. Every treated tumor was evaluated on the first study following treatment with documentation of the following post-treatment characteristics: ablation cavity size in two perpendicular measurements in maximum trans-axial dimension; presence or absence of treatment response features including presence and type of enhancement; size of viable or equivocal disease, and treatment response category based on the LIRADS TRA v2018 [5].

After explant graft data was collected, new imaging occult- treatment naïve tumors were found. One of the three readers (A.S.S.B) reviewed the immediate pre-transplant studies. The visible new observations were localized by correlating with the hepatic segment at pathology and documented using LI-RADS categories.

### Pathologic analysis

Explant pathology analysis was performed following LT at the Pathology Department of Beth Israel Deaconess Medical Center which has the experience of dealing with pathology for liver transplant for approximately 40 years. Depending on the year of transplant, staging was carried out according to the American Joint Committee on Cancer Staging Manual (7th Edition, 2009; 8th Edition, 2017) and College of American Pathologists Cancer Protocol for the Examination of Specimens from Patients with Hepatocellular Carcinoma (October 2013; June 2017) [17, 18]. All pathology reports also included staging according to the American Liver Study Group for transplant purpose. Every liver received was grossly evaluated and fixed in 10% buffered formalin. The liver was serially sectioned at 5 mm intervals. The liver parenchyma was examined and search for nodules and reconciliation with pretransplant imaging was carefully done. Nodules not described by radiology were documented and described separately. Grossly, for each of the tumor nodules, the number, location in which segment, distance from the closest margin, satellitosis and gross vascular invasion was documented. Macroscopic viability was further dichotomized as partial (5–50% viable HCC) or extensive (> 50% viable HCC).

Each nodule was extensively sampled for histopathologic examination, and depending on the size, was totally submitted for histopathological examination. Histopathological evaluation included assessment of pathologic grade, necrosis, size, microscopic vascular invasion and treatment effect when applicable. Pathologic tumor viability was reported as non-viable or completely necrotic (100% necrosis of HCC) or viable HCC (99% or less necrosis). Pathologic viability was categorized as microscopic (< 5% viable HCC) or macroscopic (> 5% viable HCC).

### Statistical analysis

Continuous variables were reported using a median and interquartile range (IQR), while categorical variables were reported as a number and percentage. Characteristics of patients, disease, and treatment were compared using t-test and Mann–Whitney U-tests for continuous variables and χ2-tests of association for categorical variables. Initial comparison included sensitivity and specificity analysis of the consensus and non-consensus sample sets. Further, in the consensus and non-consensus sample set, agreement of pre-transplant imaging and explant (viable vs. non-viable) was assessed using the Cohens Kappa test. Additionally, in the non-consensus sample set (three raters), inter-reader agreement for components of LIRADS (APHE, Washout, Capsule, Category) and LIRADS-TRA or LR-TR (APHE, Washout, Category) were assessed using the Fleiss κ test, with 95% confidence intervals obtained with the bootstrap technique. LR-TR and explant comparative analysis was carried out twice, first by keeping equivocal cases in the non-viable category and then in the viable category. Exploratory analysis included inter-reader agreement for LR-TR (pre-transplant imaging) as well as LR-TR and explant comparative analysis stratified by CT and MRI. The measurement of observer agreement for categorical data was assessed as < 0: poor agreement, 0.0-0.20: slight, 0.21–0.40: fair, 0.41–0.60: moderate, 0.61–0.80: substantial, and 0.81-1.0: almost perfect agreement [19].

## Results

### Demographic data

A total of 119 consecutive adult patients (median age 59 years [IQR: 53–64 years], 95 [80%] male) with 165 HCCs treated with thermal ablation, who underwent LT were included. All patients had cirrhosis with a majority (82 [69%]) secondary to viral hepatitis (Hepatitis B or C). Most patients had Child Pugh A cirrhosis (83 [70%]) and preserved ECOG (Eastern Cooperative Oncology Group) performance status, with score of 0–1 (116 [97%]) (Table 1).

**Table 1:** Baseline patients’ characteristics

Variables	Median [IQR] or *N* (%)
Total Patients	119
Age	59 (53–64)
Gender	
Female	24 (20%)
Male	95 (80%)
**Etiology of cirrhosis**	
Autoimmune	2 (2%)
Alcoholic Hepatitis	16 (13%)
Hemochromatosis	1 (1%)
Viral Hepatitis	82 (69%)
Viral and Alcoholic Hepatitis	9 (8%)
MASH	9 (8%)
**ECOG performance status**	
0 = Fully Active	104 (87%)
1 = Restricted in strenuous activity	12 (10%)
2 = Ambulatory	3 (3%)
**Baseline laboratory values**	
AFP	9 (4–45)
Creatinine	0.8 (0.8-1)
Total bilirubin	1 (0.7–1.7)
INR	1.2 (1.1–1.4)
Sodium	139 (137–141)
Albumin	4 (3–4)
MELD Score	10 (8–14)
**Baseline comorbidities**	
Encephalopathy	6 (5%)
Ascites	32 (27%)
Slight	18 (56%)
Moderate/Severe	14 (44%)
**CP score**	
hild Pugh A	83 (70%)
Child Pugh B	34 (28%)
Child Pugh C	2 (2%)
**HCCs on pre-treatment imaging**	165 HCCs
Patients with 1 HCC	83 (70%)
Patients with 2 HCCs	27 (23%)
Patients with 3 HCCs	8 (7%)
Patients with 4 HCCs	1 (1%)

*Abbreviations* MASH: Metabolic-dysfunction associated steatohepatitis, ECOG: Eastern Cooperative Oncology Group, AFP: alpha-fetoprotein; INR: International normalized ratio; MELD: Model for End-Stage Liver Disease; CP: Child Pugh; HCC: Hepatocellular carcinoma

### Pre-treatment imaging (LI-RADS categories)

Of the 119 patients, 78 (66%) underwent contrast-enhanced MRI and 41 (34%) underwent multiphasic CT as the initial study before treatment. Of these, 83 (70%) had a solitary HCC, 27 (23%) had 2 HCCs, 8 (7%) had 3 HCCs and 1 (1%) had 4 HCCs for a total of 165 HCCs (median size 2.2 cm [IQR: 1.7–2.5 cm]). The tumor distribution by size included 67 (41%) tumors measuring 1–2 cm; 97 (59%) measuring 2.1–5.0 cm; and only 1 (1%) measuring > 5 cm. Majority of the tumors (116 [70%]) were noted to be in the right lobe with segmental distribution using Couinaud’s segmental anatomy as shown in (Table 2). The pre-treatment LI-RADS category for 165 tumors included 136 (82%) LIRADS 5; 19 (12%) LIRADS 4 and 10 (6%) were LR M category biopsy proven HCC.

**Table 2:** Tumor distribution by Couinaud’s segments

Couinaud’s segments	Pre-treatment tumor location *N* (%)	Viable tumors on explant *N* (%)
Segment 1	1 (1%)	0 (0%)
Segment 2	8 (5%)	3 (6%)
Segment 3 or 2/3 *	10 (6%)	3 (6%)
Segment 4 A	20 (12%)	7 (13%)
Segment 4B	10 (6%)	2 (4%)
Segment 5 or 5/8**	22 (13%)	6 (11%)
Segment 6	30 (18%)	9 (17%)
Segment 7	23 (14%)	7 (13%)
Segment 8	41 (25%)	16 (30%)

* Two patients had tumor in both segment 2 and 3

** One patient had tumor in both segment 5 and 8

### Ablation cavity

A total of 142 ablation procedures were performed for 119 patients, with 101 (85%) patients undergoing 1 ablation; 14 (12%) undergoing 2 ablations; 3 (3%) undergoing 3 ablations and 1 (1%) patient undergoing 4 ablation procedures. Median ablation cavity size was 3.9 cm [IQR:3.3–4.7 cm] x 2.7 cm [IQR: 2.2–3.2 cm]. Ablation cavity sizes were further divided into 3 categories based on the maximum trans-axial dimension of the cavity. Of these, there were 2/165 (1%) ablation cavities in the 1–2 cm group; 133/165 (81%) in the 2.1–5.0 cm group, and 30/165 (18%) > 5 cm in the maximum trans axial dimension.

### Pre- transplant imaging

All patients in this study had a pre-transplant scan within 3 months of LT (median 42 days [IQR:22–68 days]). The median time between the last treatment and the pre-transplant scan was 9 months (IQR: 5–15 months). Of the 119 patients, 78 (66%) underwent a contrast-enhanced CT study and 41 (34%) underwent an MRI. A consensus read of all 3 readers to assess the *LR-TR* categories of treated tumors for all pre-transplant scans was obtained.

Of these, 147 (89%) were LR-TR non-viable; 16 (10%) were LR-TR viable and 2 (1%) treated tumors were LR-TR equivocal (Table 3). Median ablation cavity size was 3.1 cm (IQR: 2.6–3.9 cm) x 2.2 cm (IQR: 1.8–2.7 cm). Based on the maximum trans-axial dimension of the cavity, there were 7 (4%) ablation cavities in the 1–2 cm group; 143 (87%) in the 2.1–5.0 cm group, and 15 (9%) > 5 cm in the maximum trans axial dimension.

**Table 3:** Tumor imaging and explant analysis

Variables	Median [IQR] or *N* (%)
**Pre-treatment imaging (LI-RADS)**	
Time before treatment (days)	34 (21–51)
Median tumor size (cm)	2.2 (1.7–2.5)
*Tumor size groups*	
1–2 cm	67 (41%)
2.1–5 cm	97 (59%)
> 5 cm	1 (1%)
*LIRADS category*	
LIRADS 5	136 (82%)
LIRADS 4	19 (12%)
LIRADS 3	7 (4%)
LIRADS-M	3 (2%)
**Pre-transplant imaging (LR-TR)**	
Time prior to transplant (days)	42 (22–68)
Median ablation cavity size (cm)	3.9 (3.3–4.7)
*Ablation cavity size groups*	
1–2 cm	7 (4%)
2.1–5 cm	143 (87%)
> 5 cm	15 (9%)
*LR-TR category*	
Non-viable	147 (89%)
Viable	16 (10%)
Equivocal	2 (1%)
**Explant histopathological analysis**	
Treated HCCs	165
Non-viable HCCs	112 (68%)
Viable HCCs	53 (32%)
Microscopic viability (< 5%)	17 (32%)
Macroscopic viability (> 5%)	36 (68%)
Treatment naïve or imaging occult tumors	55
Median tumor size (cm)	1.0 (0.6–1.5)

*Abbreviation* LI-RADS TRA: Liver Imaging Reporting and Data System Treatment Response algorithm

### Explant pathology analysis

Of the 165 known tumors assessed on pre-treatment imaging, 112 (68%) were completely necrotic and 53 (32%) were viable on pathologic analysis (Table 3 and Figure S2). Of these, 17/53 (32%) were *≤* 5% viable at microscopy with the pre-treatment tumor size ranging from 1.2 cm to 3.6 cm (mean- 2.0 cm); 27/53 (51%) were 5–50% viable with the pre-treatment tumor size ranging in size from 1.5 cm to 3.8 cm (mean- 2.3 cm); and 9/53 (17%) of the tumors were > 50% viable at microscopy, with the pre-treatment tumor size ranging from 2.0 cm to 5.9 cm (mean- 2.8 cm).

Tumors that were viable on explant histopathology were larger on pre-treatment imaging compared to tumors that were *completely necrotic* (median tumor size 2.4 vs. 2.1 cm, *p* = 0.02). The most common location [5/17 (29%)] of the tumors that were *less than or equal to 5% viable* was segment 7 [10/27 (37%)]; of the *5–50% viable* were in segment 8 and [3/9(33%)] of those that were *more than 50% viable* were in segment 4 A. There was no difference in pre-transplant median ablation cavity sizes between viable and completely necrotic tumors (4.0 vs. 3.9 cm; *p* = 0.58).

### Comparison of pre-transplant LR-TR category to explant pathology

Following consensus read (Table 4), 16/165 tumors were called viable (10%); 147/165 (89%) were non-viable and 2/165 (1%) tumors were equivocal. Of the 16 tumors that were called viable by imaging, 10/16 (62.5%) were truly viable on explant pathology and 6/16 (37.5%) were non-viable. Of the 147/165 tumors that were called non-viable by imaging, 104/147 (71%) were truly non-viable on explant pathology and 43/147(29%) were viable.

**Table 4:** Non-consensus and consensus Pre-Transplant LR-TR vs. Explant stratified by imaging modality**

LRTR^1^ Viable vs. (Non-viable and Equivocal)																		
	Overall Cohort						CT Cohort						MRI Cohort					
	SN	SP	AC	CK	Z	*P*	SN	SP	AC	CK	Z	*P*	SN	SP	AC	CK	Z	*P*
R1	7 (13%)	105 (96%)	112 (69%)	0.12	2.26	0.02	4 (11%)	75 (95%)	79 (69%)	0.1	1.18	0.24	3 (18%)	30 (100%)	33 (70%)	0.22	2.38	0.02
R2	6 (11%)	105 (95%)	111 (68%)	0.09	1.62	0.11	2 (6%)	76 (95%)	78 (67%)	0.01	0.13	0.90	4 (24%)	29 (97%)	33 (70%)	0.24	2.16	0.03
R3	4 (8%)	108 (98%)	112 (69%)	0.07	1.82	0.07	1 (3%)	78 (98%)	79 (68%)	0.004	0.09	0.93	3 (18%)	30 (100%)	33 (70%)	0.22	2.38	0.02
Con	10 (19%)	106 (95%)	116 (70%)	0.17	2.74	0.01	4 (11%)	76 (94%)	80 (68%)	0.1	0.93	0.36	6 (35%)	30 (97%)	36 (75%)	0.37	3.01	0.003
**LRTR** ^**1**^ **Non-viable vs. (Viable and Equivocal)**																		
R1	9 (17%)	105 (96%)	114 (70%)	0.17	2.93	0.003	4 (11%)	75 (95%)	79 (69%)	0.1	1.18	0.24	5 (29%)	30 (100%)	35 (74%)	0.35	3.14	0.002
R2	6 (11%)	105 (95%)	111 (68%)	0.09	1.62	0.11	2 (6%)	76 (95%)	78 (67%)	0.01	0.13	0.90	4 (24%)	29 (97%)	33 (70%)	0.24	2.16	0.03
R3	8 (15%)	105 (95%)	113 (69%)	0.13	2.33	0.02	4 (11%)	76 (95%)	80 (69%)	0.1	1.20	0.23	4 (24%)	29 (97%)	33 (70%)	0.24	2.16	0.03
Con	10 (19%)	104 (93%)	114 (69%)	0.14	2.26	0.02	4 (11%)	75 (93%)	79 (68%)	0.05	0.66	0.51	6 (35%)	29 (94%)	35 (73%)	0.33	2.56	0.01

^1^CT/MRI LI-RADS^®^ v2018 CORE

**Abbreviations.** LRTR: locoregional treatment response; SN: sensitivity; SP: specificity; AC: accuracy; CK: Cohens Kappa; Z: z-value; P: p-value; R1: reader 1; R2: reader 2; R3: reader 3; Con: consensus pool; CT: cat-scan; MRI: magnetic resonance imaging

*Number of observations - ***Overall cohort***: Consensus (165), Nonconsensus (R1: 163; R2: 163; R3: 163); ***CT cohort***: Consensus (117), Nonconsensus (R1: 115; R2: 116; R3: 116); ***MRI cohort***: Consensus (48), Nonconsensus (R1: 47; R2: 47; R3: 47)

Positive predictive value (PPV) for viable tumor was 62.5% (CI 39–81%) (*p* = 0.01). Negative predictive value for non-viable tumor at pathology was 38% (CI 19–61%) (*p* = 0.02). Sensitivity to detect viable tumor at pathology was 19% (CI: 9.4–32%); specificity was 95% (CI: 89–98%) and accuracy was 70% (CI: 63–77%). Sensitivity to detect non-viable tumor at pathology was 19% (CI: 5–25%); specificity was 93% (CI: 85–96%) with an accuracy of 69% (CI: 60–75%). Only 2 tumors were reported as equivocal and both were found to be completely necrotic at pathology, hence were included as non-viable for the purpose of calculation. [Figure S3].

Treatment *naïve or imaging occult tumors on explant*: There were 55 new treatment-naïve tumors (median size 1 cm, IQR: 0.6–1.5 cm) found on explant pathology in 33/119 (28%) patients that were imaging occult on the pre-transplant scans at the first round of image interpretation. Of these, 27/55 (49%) tumors were less than 1 cm; 25/55 (45%) tumors were 1–2 cm in size, and 3/55 (5%) tumors were more than 2 cm in size. Majority (42/55 [76%]) of the imaging occult tumors were present in the right lobe.

The senior-most radiologist (A.S.S.B), blinded to patient and tumor data, took a directed second look at all the pre-transplant scans to assess for these occult tumors (Table S1). On second look on pre-transplant scans, 42 lesions presumed to be HCC (median size 1.2 cm, IQR: 0.9–1.5 cm) were identified in 28/119 (24%) patients. These were identified based on the location specified in the explant but did not meet all imaging criteria for LIRADS category 4 or 5 observation.

**Fig. 1:**
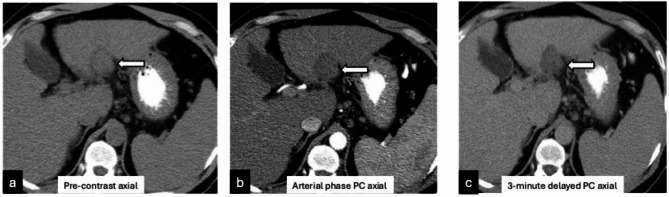
44-year-old male with segment II hepatocellular carcinoma treated with radiofrequency ablation. One month follow up pre-contrast (**a**) and post-contrast (**b**-**c**) CT imaging demonstrates no suspicious arterial hyperenhancement (b, arrow) or washout (c, arrow) suggesting LR-TR nonviable observation. At explant histopathology, this lesion was noted to contain 5–50% viable tumor

**Fig. 2:**
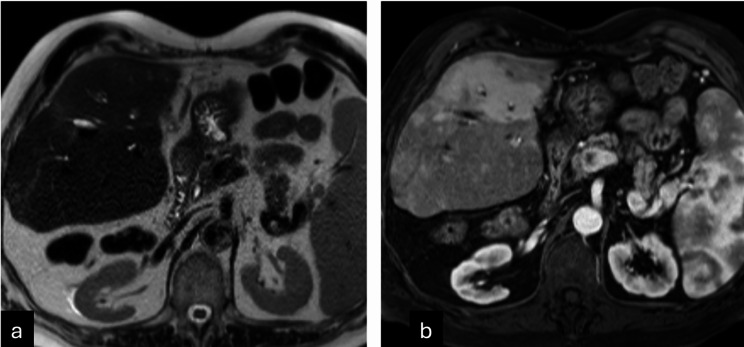
64-year-old male with a 1.3 cm treatment naïve new hepatocellular carcinoma was found on explant histology, located in segment V. T2 axial (**a**) and arterial phase T1-post-contrast axial (**b**) images on MRI performed one month prior to transplant demonstrates no focal lesion suspicious for HCC, suggesting an imaging occult tumor

**Fig. 3:**
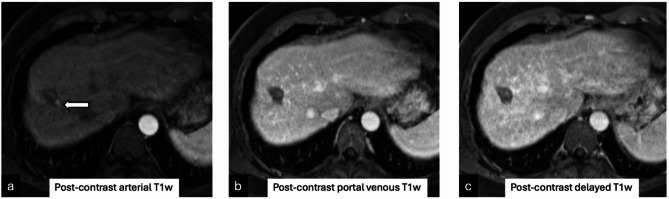
59-year-old male with a 1.8 cm LIRADS-5 observation at the hepatic dome in segment VIII treated with microwave ablation, demonstrates a peripheral enhancing nodule on the arterial phase (**a**, arrow); that is not conspicuous on the portal venous or delayed phase (**b**,**c**) on the pre-transplant multiphase MRI, interpreted as LR-TR viable. Complete pathologic necrosis was seen within this tumor at explant histopathology

Examples of LR-TR nonviable observation on post-treatment imaging with microscopy demonstrating 5–50% viable tumor (Figure 1); 1.3 cm treatment naïve new HCC noted on explant histology but occult on pre-transplant imaging (Figure 2); and tumor interpreted as LR-TR viable on pre-transplant imaging demonstrating complete pathologic necrosis at histology are demonstrated (Figure 3).

### Non-consensus and consensus pre-transplant LR-TR vs. explant stratified by imaging modality

In the consensus pool, the degree of objective agreement between LR-TR on immediate pre-transplant imaging and explant analysis for the entire cohort ranged from 0.14 to 0.17 using Cohens Kappa (SN 19%, SP 93–95%, Acc 69–70%), and was numerically higher among the MRI cohort with Cohens Kappa of 0.33–0.37 (SN 35%, SP 94–97%, Acc 73–75%) vs. the CT cohort with Cohens Kappa of 0.004-0.8 (SN 11%, SP 93–94%, Acc 68%). A similar trend of higher objective agreement as well as higher sensitivity, specificity, and accuracy was noted in the non-consensus pool for the MRI cohort compared to the CT cohort (Table 5).

**Table 5:** Comparison of pre-transplant interpretation with explant pathology stratified by patients with and without viable tumor on explant

Variables	Sensitivity (%)	Specificity (%)	Accuracy (%)	PPV (%)	NPV (%)	Cohens Kappa	*P*-value
Viable tumor at pathology (*n* = 53)	10 (19%) [9.4–32]	106 (95%) [89–98]	116 (70%) [63–77]	62.5% [39–81]	71% [68–74]	**0.2**	0.01
Non-viable tumor at pathology (*n* = 112)	10 (19%) [5–25]	104 (93%) [85–96]	114(69%) [60–75]	71% [69–73]	38% [19–61]	**0.1**	0.02

*Abbreviations* PPV: Positive predictive value, NPV: Negative predictive value

While observed agreement between readers was high across CT and MRI (ranging from 77 to 91%), Fleiss’ Kappa values, however, ranged from 0.13 to 0.47, indicating fair to moderate agreement after adjusting for chance. Agreement was generally higher for CT (e.g., category K = 0.47) and lower for MRI (e.g., category K = 0.31), suggesting more consistent scoring on CT compared to MRI (Table 6).

**Table 6:** Inter-reader agreement using kappa Fleiss for LR-TR (pre-transplant imaging) stratified by modality

	Overall			CT			MRI		
	APHE	Washout	LR-TR Category	APHE	Washout	LR-TR Category	APHE	Washout	LR-TR Category
Observed Agreement	86%	91%	88%	89%	91%	91%	77%	89%	81%
Number of Entries	161	160	162	114	114	115	47	46	47
Number of Raters	3	3	3	3	3	3	3	3	3
Fleiss K	0.39	0.37	0.41	0.46	0.44	0.47	0.26	0.13	0.31
CI	0.17–0.56	0.11–0.56	0.18–0.59	0.16–0.69	0.13–0.70	0.14–0.72	-0.02-0.55	-0.05-0.38	0.01–0.56

*Abbreviations* APHE: arterial phase hyperenhancement

## Discussion

This is a retrospective study of HCC patients treated with percutaneous thermal ablation prior to liver transplantation, with post treatment imaging reads at multiple time points by 3 readers and consensus reads used to determine treatment response, compared to explant pathology. The overall CPN rate (68%) at this center compares favorably with the median center-specific CPN rate (25%) in US transplant centers [14]. However, there was low sensitivity [19%] and high specificity [95%] for the viable category with an accuracy of 70%. Explant pathology revealed 55 new treatment naïve tumors, missed on imaging, in 33 patients. The median size of the missed tumors was 1.0 cm, but 3 tumors had a maximum diameter > 2 cm.

The low sensitivity and high specificity of the LI-RADS treatment response algorithm (TRA) (v2018) noted in our study has been previously reported [10, 13]. However, several factors may result in this finding being more pronounced in the current study compared to previously published literature. Majority of the imaging examinations were performed using multiphasic CT (66%) versus MRI (34%), and MRIs were performed entirely with extra-cellular (rather than hepatobiliary) contrast. The sensitivity for detecting viable tumor was lower (19%) when compared to the pooled sensitivity in recent prior meta-analyses, that were in the range of 56–63% [13, 12]. Compared to prior studies, this may be due to less experienced readers (all 3 readers with less than 9 years of post-fellowship experience), the higher utilization of CT for pre-transplant imaging, or that all patients in the current study were treated with thermal ablation, which has been identified as a factor for lower sensitivity for viable tumor in a meta-analysis [13]. These findings suggest that further refinement and standardization of the imaging technique and development of imaging tools that are less operator- and treatment modality-dependent are necessary in the assessment of viable tumor on imaging prior to LT.

Prior papers have attempted to improve the sensitivity further by including the LR-TR equivocal category in the viable category (assuming that equivocal were viable) and demonstrated improved sensitivity from 63 to 71% [13, 12] and from 81 to 87% [8]. However, the small number of equivocal category tumors in this study did not change the sensitivity when LR-TR viable and equivocal were combined due to the less frequent use of the equivocal category tumors and the fact that both equivocal tumors were non-viable at pathology. This finding contradicts the presumption that most equivocal category tumors have residual viable disease and are incompletely necrotic as shown previously by Chaudhry et al. [6/53 equivocal; 5 viable] [8]; by Shropshire et al. [17/63 equivocal; 12 viable] [9]; Seo et al. with viability in equivocal lesions ranging from 50 to 100% [11].

We used the same binary system as Shropshire and Hassan [9, 10] to define tumor response at explant pathology i.e., non-viable if 100% necrotic and viable if 99% or less necrotic. However, one issue affecting the generalizability and reproducibility of rad-path correlations is that while the imaging techniques and interpretations are standardized across multiple readers, existing studies do not report a uniform method for assessing complete pathological response. In this study, viable disease was categorized into microscopic (*≤* 5% viable HCC) or macroscopic disease (> 5% viable HCC). Macroscopic viability was further dichotomized as partial (5–50% viable HCC) or extensive (> 50% viable HCC). There was no significant difference in pre-treatment tumor size between the *≤* 5% and 5–50% viable HCC categories with a slightly larger average pre-treatment tumor size of 3.0 cm in the > 50% viable category. A prior study has shown about 67% of pathologically viable tumor to be more than 1.0 cm in size [20]. However, it raises the question regarding the significance of viable tumor less than 1 cm or even smaller in size. Encouragingly, in a study of 5242 patients, Mahmud et al. [21] found that lesions less than 1.0 cm in size did not contribute to recurrence or mortality following transplant. Future studies that standardize the assessment and categorization of resected or transplanted liver specimens with respect to complete and incomplete pathological response are warranted and necessary.

Additional HCC lesions, missed on pre-transplant imaging, within the explanted liver is also a source of disease under-staging, potentially leading to post-transplant HCC recurrence and worse outcomes [21- 25]. The reported incidence ranges from 20% [24] to 33.3% [23], with this study noting an incidence of 28% of incidental additional tumors on explant pathology. Reasons cited in literature for non-detection or under-staging of tumors on pre-transplant studies range from poor visibility of small tumors in a cirrhotic liver [24], multiple tumors > or = 2 tumors [26], inability of imaging studies to detect microvascular tumor invasion [27], post-liver directed therapy altering ability to accurately detect viable disease [28], with a reported 1.85-fold increased odds of under-staging following ablation relative to chemoembolization reported in one study [21]. One of the readers (A.S.S.B) re-evaluated the pre-treatment scans knowing segmental location of these tumors from explant data and detected 24% of these tumors that were missed on the first round of interpretation. Reasons for missing these tumors at initial reads were deliberately sought and included tumor location at the cranial most aspect of the hepatic dome, caudate lobe and inferior tip of the liver; inadequate windowing on arterial phase compounded by too early or too late an arterial phase resulting in suboptimal arterial phase hyper-enhancement of the tumor; diffuse hepatic fibrosis in a reticular pattern limiting visualization of washout especially for small tumors; non diagnostic quality diffusion weighted sequences at the hepatic dome and inferior aspect of the liver; tumors close to larger portal venous branches obscured by adjacent contrast and misinterpretation of nodular enhancement in the treatment bed either due to obscuration by surrounding perfusional changes or due to lack of comparison with pre-treatment scan leading to non-recognition of residual tumor masked by post-treatment changes.

Our study limitations included its retrospective nature. Of the 119 patients, 114 were scanned on the 64-Slice scanner with 5 patients being scanned on a 16-Slice scanner prior to 2004, however, the multi-phase scan protocol satisfied the technical requirements established by LI-RADS obviating the need to exclude these patients. The low number of equivocal tumors may reflect potential reader overconfidence in using the TRA. Similar to the recent paper by Hassan et al. [10], due to lack of a standardized definition of percentage tumor necrosis that is clinically significant, we used a binary system to determine complete pathologic necrosis. We did subclassify pathologic necrosis into three additional subgroups, a system that remains to be validated by additional studies.

## Conclusion

In conclusion, our study reinforces the low to moderate sensitivity, albeit with high specificity, of the LI-RADS TRA “non-viable” category in assessment of true pathologically viable HCC in patients treated by non-radiation therapy. Specifically, imaging misclassified viable lesions as nonviable as well as missed new small HCC lesions, resulting in missed opportunities for excluding patients with tumor burden at higher risk for recurrence post-transplant and/or additional locoregional therapy. Pre-transplant imaging stratified by imaging modality demonstrated that MRI had more accuracy than CT in predicting tumor viability when compared to explant pathology. The clinical relevance of this data is that it highlights the need for an alternative name to the “non-viable” category in the LI-RADS lexicon that would truly represent the limitation of current cross-sectional imaging modalities in identifying pathologically viable HCC on pre-transplant scans and demonstrates the importance of utilizing MRI as a pre-transplant imaging modality over a multiphase CT to improve accuracy of lesion categorization.

## Electronic supplementary material

Below is the link to the electronic supplementary material.

Supplementary Material 1

## Data Availability

No datasets were generated or analysed during the current study.

## Abbreviations

HCC: Hepatocellular Carcinoma
LI-RADS: Liver Imaging Reporting and Data Systems
TRA: Treatment Response Algorithm
APHE: Arterial Phase Hyperenhancement
LT: Liver Transplantation
LRT: Locoregional Therapy
CPN: Complete Pathologic Necrosis
LR-TR: Liver Imaging Reporting and Data Systems Treatment Response
MRI: Magnetic Resonance Imaging
UNOS: United Network for Organ Sharing
ECOG: Eastern Cooperative Oncology Group
CP: Child- Pugh
RFA: Radiofrequency Ablation
MWA: Microwave Ablation
CLIA-88: Clinical Laboratory Improvement Amendments of 1988
IQR: Interquartile range
MASH: Metabolic dysfunction-associated Steatohepatitis
AFP: Alpha Fetoprotein
INR: International Normalized Ratio
MELD: Model for End-Stage Liver Disease
SSFSE: Single Shot Fast-Spin Echo
DWI: Diffusion Weighted Imaging
LAVA: Liver acceleration volume acquisition
