# Supplementary material for: Accuracy of cross-sectional imaging in predicting tumor viability using the LI-RADS treatment response algorithm after image-guided percutaneous ablation with radiologic-pathologic explant correlation
Source: Cancer Imaging. 2025 May 24;25:65. doi: 10.1186/s40644-025-00884-y (PMC12103036; doi:10.1186/s40644-025-00884-y)
Supplement: Supplementary file 1 — Supplementary Material 1 [file 40644_2025_884_MOESM1_ESM.docx]

**Supplemental Methods:**

**MRI Protocol:**

MRI examinations were performed on either a 1.5T (Magnetom Avanto or Aera, Siemens

Healthcare) or 3T (Magnetom Trio or Skyra, Siemens Healthcare) MRI system using a torso phased array coil. All MRI examinations included a standard abdominal MRI protocol

Including a 3 Plane localizer (SSFSE); Coronal SSFSE (through the entire liver); Axial DWI (b50, b600); Axial T1 in/out phase; Axial SSFSE; Axial 3D LAVA XV pre-contrast followed by Axial 3D LAVA XV postcontrast arterial [45 sec after arterial], venous [90 sec after arterial] and delayed [135 sec after arterial] phases.

*(Abbreviations. SSFSE: Single shot fast spin echo; DWI: diffusion weighted imaging; LAVA: liver acceleration volume acquisition)*.

**Table S1: Second look of pre-transplant scan for treatment naïve tumors found on explant pathology.**

|  | | **Explant (imaging occult tumors)** | | | | |  |
| --- | --- | --- | --- | --- | --- | --- | --- |
|  |  | **0 tumor** | **1 tumor** | **2 tumors** | **3 tumors** | **7 tumors** | **Total patients** |
| **2^nd^ read on Pre-transplant Imaging** | **0 tumors presumed** | 77 (90%) | 8 (44%) | 6 (50%) | 0 (0%) | 0 (0%) | **91** |
|  | **1 tumor presumed** | 8 (9%) | 8 (44%) | 1 (8%) | 1 (50%) | 1 (100%) | **19** |
|  | **2 tumors presumed** | 1 (1%) | 0 (0%) | 4 (33%) | 0 (0%) | 0 (0%) | **5** |
|  | **3 tumors presumed** | 0 (0%) | 1 (6%) | 1 (8%) | 1 (50%) | 0 (0%) | **3** |
|  | **4 tumors presumed** | 0 (0%) | 1 (6%) | 0 (0%) | 0 (0%) | 0 (0%) | **1** |
|  | **Grand Total** | **86 patients** | **18 patients** | **12 patients** | **2 patients** | **1 patient** | **119 patients** |

**Figure S1.**

**

**

Abbreviations. HCC: Hepatocellular carcinoma, TACE: Trans-arterial chemoembolization, Y-90: Yttrium-90.

**Figure S2.** Descriptive Tumor statistics viability on pre-transplant imaging and explant analysis.

**

**

Abbreviation: LR-TR: Liver Imaging Reporting and Data Systems Treatment Response

**Figure S3.** Tree-based distribution of pre-transplant LR-TR with explant and tumor viability. Red boxes: LR-TR; Green boxes: explant; Yellow boxes: tumor viability.


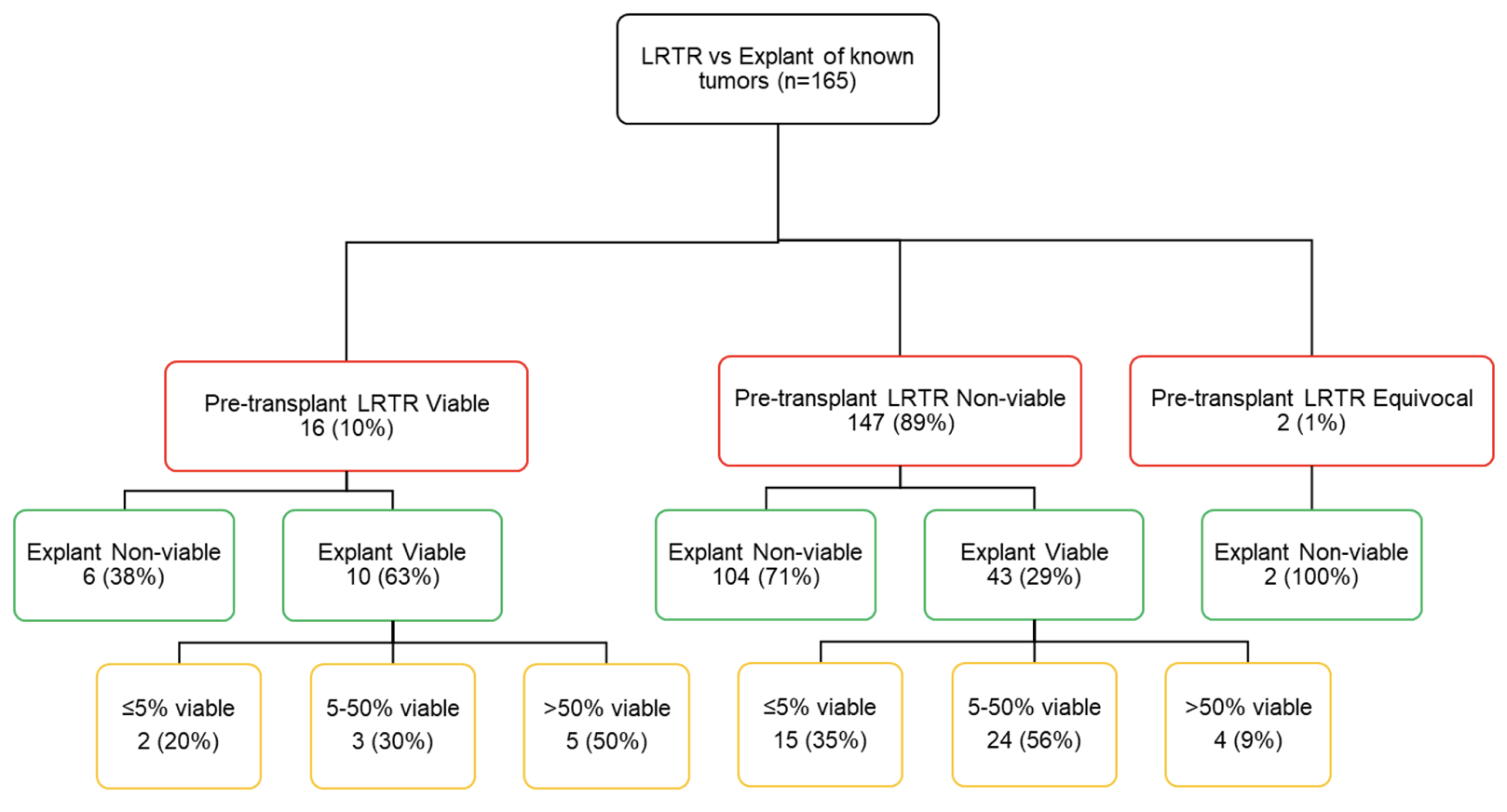


Abbreviation: LR-TR: Liver Imaging Reporting and Data Systems Treatment Response
